# Carbon monoxide poisoning surveillance in the Veterans Health Administration, 2010–2017

**DOI:** 10.1186/s12889-019-6505-y

**Published:** 2019-02-14

**Authors:** Gina Oda, Russell Ryono, Cynthia Lucero-Obusan, Patricia Schirmer, Mark Holodniy

**Affiliations:** 1 Public Health Surveillance and Research, Department of Veterans Affairs, 3801 Miranda Avenue (132), Palo Alto, CA 94304 USA; 2 Division of Infectious Diseases and Geographic Medicine, Stanford University, Stanford, CA USA

**Keywords:** Carbon monoxide poisoning, Veterans health, Public health, Surveillance, Epidemiology, Toxicology, Environmental exposures

## Abstract

**Background:**

Exposure to carbon monoxide (CO), the odorless, colorless gas resulting from incomplete combustion of hydrocarbons, is preventable. Despite the significant risk of morbidity and mortality associated with CO poisoning, there currently exists no active national CO surveillance system in the United States (U.S.). Our study aims to use electronic health record data to describe the epidemiology of CO poisoning in the Veterans Health Administration healthcare population.

**Methods:**

We identified unique inpatient and outpatient encounters coded with International Classification of Diseases (ICD) codes for CO poisoning and analyzed relevant demographic, laboratory, treatment, and death data from January 2010 through December 2017 for Veterans across all 50 U.S. states and Puerto Rico. Statistical methods used were 95% CI calculations and the two-tailed z test for proportions.

**Results:**

We identified 5491 unique patients with CO poisoning, of which 1755 (32%) were confirmed/probable and 3736 (68%) were suspected. Unintentional poisoning was most common (72.9%) overall. Age less than 65 years, residence in Midwest U.S. Census region versus South or West, and winter seasonal trend were characteristics associated with confirmed/probable CO poisoning. Twenty-six deaths (1.5%) occurred within 30 days of confirmed/probable CO poisoning and were primarily caused by cardiovascular events (42%) or anoxic encephalopathy (15%).

**Conclusions:**

Our findings support the use of ICD-coded data for targeted CO poisoning surveillance, however, improvements are needed in ICD coding to reduce the percentage of cases coded with unknown injury intent and/or CO poisoning source. Prevalence of CO poisoning among Veterans is consistent with other U.S. estimates. Since most cases are unintentional, opportunities exist for provider and patient education to reduce risk.

**Electronic supplementary material:**

The online version of this article (10.1186/s12889-019-6505-y) contains supplementary material, which is available to authorized users.

## Background

Exposure to carbon monoxide (CO), the odorless, colorless gas resulting from incomplete combustion of hydrocarbons, is avoidable. However, CO poisoning is one of the most common causes of unintentional poisoning deaths in the U.S. [1, 2] It is estimated that unintentional, non-fire related (UNFR) CO poisoning causes an average of 438 deaths annually. In 2007, confirmed CO poisoning cases resulted in 21,304 U.S. emergency department (ED) visits and 2302 hospitalizations (71 per million and 8 per million population, respectively) [3]. However, other analyses estimate that over 50,000 ED visits for CO poisoning occur annually, which may still be an underestimate because the condition is underdiagnosed [4]. Data reported to the National Poison Data System estimates that UNFR CO poisoning exposures occur at a rate of 23.2 per million population per year [1]. Despite the significant risk of morbidity and mortality associated with CO poisoning, there currently exists no active national CO surveillance system in the U.S. [5] Studies utilizing administrative data for case-finding generally do not include more current International Classification of Diseases, 10th revision, Clinical Modification (ICD-10-CM) coded data for comparison ICD-9-CM-based estimates [6, 7].

Limited data exist on population risk factors that predispose to CO poisoning. Additionally, CO poisoning as a suicide method is not well studied. Few local/state-based CO poisoning surveillance systems include intentional CO poisonings [8–10], and data examined are often limited to post-mortem datasets such as the National Death Index or state Medical Examiner records. Therefore, a complete understanding of CO poisoning-associated suicide attempts is lacking.

The Veterans Health Administration (VHA), within the Department of Veterans Affairs (VA) is the largest integrated health care system in the U.S., providing care to over 9 million enrolled Veterans at 172 hospitals and 1062 outpatient sites across all 50 states, District of Columbia, and U.S. territories [11]. Veterans are at risk for smoking and cardiovascular disease [12, 13] which could place them at higher risk for adverse outcomes from CO poisoning [14]. Veterans are also at increased risk for suicide [15, 16], however, CO poisoning as a suicide method in this population has not been studied. Sharp decreases occurred among the general population in CO poisoning suicides associated with motor vehicle exhaust since the introduction of stricter automobile emissions [17, 18]. Given that CO poisoning causes serious morbidity and mortality, a better understanding of CO poisoning trends among Veterans is critical. The goal of this study is to describe the epidemiology of CO poisoning among Veterans cared for in VHA using national integrated electronic health record data.

## Methods

### Study population

We analyzed VA electronic medical record (EMR) data for inpatient discharges and outpatient encounters from January 2010 through December 2017 with ICD-9-CM or ICD-10-CM codes meeting the 2018 Council of State and Territorial Epidemiologists (CSTE) criteria for CO poisoning case ascertainment using administrative data [19]. Veterans receiving care at non-VA healthcare facilities during the study period and whose coded encounters met CSTE criteria for CO poisoning were included. Patients with multiple visits classified as CO poisoning during the study period were counted once, for their initial encounter. Demographic, laboratory, and treatment data, and date of death (where applicable) recorded in the EMR for each unique patient with CO poisoning for the study time-period was collected. CO poisoning surveillance was conducted as part of VHA public health operations. As such, the Veterans Health Administration Office of Research Oversight considers public health investigations as operational activities and not research in VHA [20].

If patients had outpatient and inpatient codes for the same day, one unique encounter was counted, with highest level of care (i.e., inpatient) selected. To reduce inclusion of patients whose ICD-9-CM code was not indicative of initial exposure, we reviewed patient problem lists and removed 34 patients whose coded problem list indicated exposure to CO occurred prior to the study period. For patient encounters with ICD-10-CM codes, we reviewed the seventh character extension to determine if the encounter was deemed initial, subsequent, or related to CO poisoning sequela and a query was performed to determine if any of these patients had CO poisoning-coded visits prior to 2010. One patient with an ICD-10-CM sequela episode of care extension had an initial encounter in 2004 and was removed.

Based on 2018 CSTE criteria utilizing ICD-9-CM and ICD-10-CM codes, unique patients were classified by degree of certainty that poisoning was CO-related (confirmed, probable, suspected). A case was considered confirmed if the inpatient discharge or outpatient encounter was coded with ICD-9-CM code 986 or ICD-10-CM code T58 (toxic effect of carbon monoxide); or with an ICD-9-CM External Cause of Injury code (E-code) explicitly indicating CO exposure was present in the absence of code 986 (E868.3, E868.8, E868.9, E952.1, E982.1). Probable cases were defined as any encounter with ICD-9-CM E-code indicating motor vehicle exhaust exposure (E868.2, E952.0, E982.0). Cases were further categorized by intent as unintentional, assault, intentional self-harm, or undetermined based upon ICD-9-CM E-codes or specific ICD-10-CM codes pertaining to the nature of CO poisoning exposure. The full list of CSTE ICD-9-CM and ICD-10-CM codes and frequency of occurrence among cases are listed in Additional file 1; Table S1.

We utilized Logical Observation Identifiers Names and Codes (LOINC) listed by the Public Health Information Network Vocabulary Access and Distribution System [21] as laboratory tests associated with CO poisoning (32160–4, 31157–1, 20563–3, 2030–5, 2031–3, 41648–7, 2032–1, and 2029–7) to determine whether carboxyhemoglobin (COHb) blood testing of patients coded for CO poisoning by ICD-9-CM or ICD-10-CM was performed. A COHb measurement was considered collected at time of encounter if the date of COHb sample collection was within 0–2 days before or after encounter date. 2018 CSTE laboratory criteria for confirmed/probable classification was based on COHb blood level > 9% for probable or > 12% for confirmed cases in settings where smoking history is unknown [19]. We analyzed procedure codes ICD-9-CM 93.95 and the ICD-10 Procedure Coding System codes 5A05121 and 5A05221 for hyperbaric oxygen (HBO_2_) therapy and dates of death within intervals of 30 days or 1 year relative to CO poisoning encounter dates as outcome data for patients with confirmed, probable, and suspected CO poisoning.

### Statistical analysis

We analyzed demographic variables and compared to unique users of VHA care during the 2010–2017 time-period, calculating 95% CIs. Seasonality trends for CO poisoning encounters were analyzed across the study period. Rates were calculated using total unique users of VHA care for matching time frame and VHA-defined demographic category as denominator. Rates of CO poisoning by U.S. Census region [22] were determined based on state location of the parent facility where the encounter occurred, using number of unique users of VHA care per state*.* Rates for confirmed/probable cases were compared to those of suspected cases across all variables looking for potential differences based on case categorization and testing for statistical significance using the two-tailed z test for proportions. Statistical analysis was performed using Python Statsmodels module, version 0.9.0 [23].

We performed EMR review for 678 patients including a) all 595 confirmed/probable CO poisoning cases from 2010 to 2016 initially categorized with undetermined injury intent; b) a random sample of 20 patients out of 1847 (1%) with suspected CO poisoning coded with ICD-9-CM code E825.x for “Other motor vehicle non-traffic accident of other and unspecified nature”; c) a random sample of 20 confirmed/probable cases with source of CO poisoning “other or unspecified”, d) all 26 patients with confirmed/probable CO poisoning who died within 30 days of CO poisoning encounter; and e) all 17 patients who received HBO_2_ therapy.

## Results

We identified 5491 unique patients with ICD-9-CM or ICD-10-CM encounter codes that met the 2018 CSTE criteria for confirmed, probable, or suspected CO poisoning. A total of 1755 (32%) were confirmed/probable cases, while 3736 (68%) were suspected. The average annual incidence rate for confirmed/probable CO poisoning was 3.6 per 100,000 unique users of VHA care (range: 3.0–4.2 per 100,000 unique users across all years, with highest at 2010 and lowest at 2017).

**Fig. 1:**
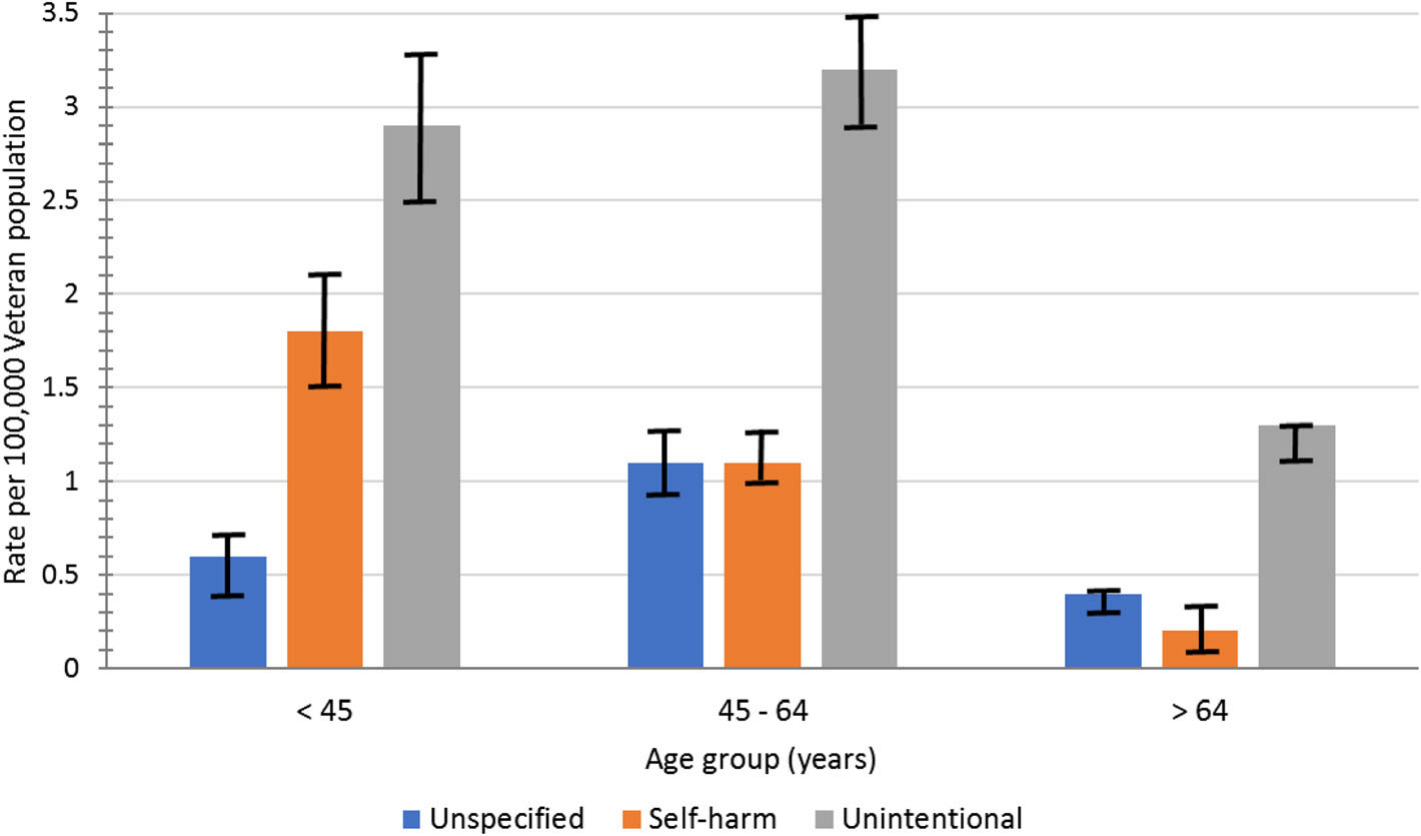
Confirmed/probable carbon monoxide poisoning rates with 95% CI by injury intent and age group, 2010 - 2017. Note: Assault injury intent not included in calculations due to case numbers less than 10 per category

Table 1 displays CO poisoning case characteristics, comparing confirmed/probable to suspected. Among the 1755 confirmed/probable cases, 746 (42.5%) had unintentional injury intent, 1274 (72.6%) were outpatient, 1556 (88.7%), were male, 883 (50.3%) were 45–64 years old, and 1286 (73.3%) were white. The source of CO poisoning was “other or unspecified” for 1289 (73.4%) of confirmed/probable cases based on associated ICD-9-CM or ICD-10-CM codes (Additional file 1). Motor vehicle exhaust was the second most common source of confirmed/probable cases, (261 cases; 14.9%). Less frequent sources among confirmed/probable cases were domestic fuel (101 cases; 5.8%), utility gas (69 cases; 3.9%), smoke/fumes from conflagration (7 cases; 0.4%), and liquefied petroleum gas (6 cases; 0.3%). Table 2 shows demographic characteristics of patients with confirmed/probable encounters compared to the overall population of unique VHA care users. Demographic factors with highest confirmed/probable CO poisoning rates were age groups < 44 (5.3 per 100,000; 95% CI = 4.8, 5.8) and 45–64 (5.4 per 100,000; 95% CI = 5.1, 5.8) and residence in the Midwest (5.3 per 100,000; 95% CI = 5.8, 5.8) compared to South or West (2.4 per 100,000; 95% CI = 2.2, 2.7 and 3.5 per 100,000; 95% CI = 3.1, 3.9, respectively). Figure 1 illustrates higher rates of confirmed/probable CO poisoning associated with self-harm in younger age groups compared to the oldest. Confirmed/probable cases did not differ from unique users of VHA care in gender or race/ethnicity.

**Table 1:** Characteristics of Veterans with confirmed/probable vs. suspected carbon monoxide poisoning, 2010–2017

Characteristic	Confirmed and Probable *N* = 1755	Suspected ^a^ *N* = 3736	*p-*value ^b^	Combined *N* = 5491
	No. (%)	No. (%)		No. (%)
Injury Category:				
Unintentional	746 (42.5)	3259 (87.2)	< 0.001	4005 (72.9)
Assault	10 (0.6)	19 (0.5)	0.77	29 (0.5)
Intentional Self-Harm	349 (19.9)	222 (5.9)	< 0.001	571 (10.4)
Undetermined	650 (37)	236 (6.3)	< 0.001	886 (16.1)
Encounter setting:				
Inpatient	458 (26.1)	277 (7.4)	< 0.001	735 (13.4)
Non-VA	23 (1.3)	175 (4.7)	< 0.001	198 (3.6)
Outpatient	1274 (72.6)	3284 (87.9)	< 0.001	4558 (83)
Gender:				
Female	199 (11.3)	419 (11.2)	0.89	618 (11.3)
Male	1556 (88.7)	3317 (88.8)	0.89	4873 (88.7)
Age groups (years):				
< 44	429 (24.4)	1077 (28.8)	< 0.001	1506 (27.4)
45–64	883 (50.3)	1704 (45.6)	0.001	2587 (47.1)
> 65	443 (25.2)	955 (25.6)	0.80	1398 (25.5)
Race/Ethnicity:				
AI/AN	11 (0.6)	29 (0.8)	0.54	40 (0.7)
Asian	6 (0.3)	25 (0.7)	0.13	31 (0.6)
Black	253 (14.4)	538 (14.4)	0.99	791 (14.4)
Hispanic/Latino	67 (3.8)	188 (5)	0.05	255 (4.6)
Multi-racial	19 (1.1)	18 (0.5)	0.01	37 (0.4)
NH/OPI	12 (0.7)	33 (0.9)	0.44	45 (0.8)
Unknown	101 (5.8)	329 (8.8)	< 0.001	430 (7.8)
White	1286 (73.3)	2576 (69)	0.001	3862 (70.3)
%COHb blood level				
Present at time of encounter ^b^	668 (38)	182 (4.9)	< 0.001	850 (15.5)
Blood level > 9% ^c^	206 (30.8)	8 (4.4)	< 0.001	214 (25.2)
Death				
Died within 30 days of encounter	26 (1.5)	2 (0.1)	< 0.001	28 (0.5)
Died within 1 year of encounter	89 (5.1)	11 (0.3)	< 0.001	100 (1.8)

*Note*: AI/AN, American Indian/Alaskan Native; NH/OPI, Native Hawaiian/Other Pacific Islander; COHb, carboxyhemoglobin

^a^Suspected cases (based on ICD-9-CM codes only) did not occur after September 30, 2015 when coding system changed to ICD-10-CM

^b^Performed same day, or 1–2 days before or after date of CO poisoning coded encounter. If more than one test was performed per unique patient, highest blood level measurement was used

^c^For subgroup of patients with carboxyhemoglobin level present at time of encounter, *N* = 850

**Table 2:** Demographic characteristics of Veterans with confirmed/probable carbon monoxide poisoning compared to all Veterans patients, 2010–2017

Characteristic	Confirmed and Probable cases		Unique users of VHA care No. users. (%)
	No. cases (%)	Rate per 100,000 unique users of care (95% CI)	
Overall	1755 (100.0)	3.6 (3.5, 3.8)	48,461,554 (100.0)
Gender:			
Female	199 (11.3)	4.2 (3.7, 4.8)	4,692,679 (9.7)
Male	1556 (88.7)	3.6 (3.4, 3.7)	43,664,749 (90.1)
Unknown	0	--^a^	104,126 (0.2)
Age groups (years):			
< 44	429 (24.4)	5.3 (4.8, 5.8)	8,131,072 (16.8)
45–64	883 (50.3)	5.4 (5.1, 5.8)	16,219,636 (33.5)
> 65	443 (25.2)	1.8 (1.7, 2.0)	24,078,923 (49.7)
Unknown	0	--^a^	31,923 (0.1)
Race/Ethnicity: ^b^			
AI/AN	8 (0.4)	--^a^	167,952 (0.3)
Asian	5 (0.3)	--^a^	254,426 (0.5)
Black	156 (8.9)	3.4 (2.9, 4.0)	4,555,304 (9.4)
Hispanic/Latino	53 (3.0)	3.0 (2.2, 3.9)	1,746,941 (3.6)
Multi-racial	8 (0.4)	--^a^	308,867 (0.6)
NH/OPI	9 (0.5)	--^a^	180,193 (0.4)
Unknown	66 (3.8)	1.6 (1.2, 2.0)	4,168,179 (8.6)
White	764 (43.5)	3.9 (3.7, 4.2)	19,427,646 (40.1)
			*N* = 30,809,508
U.S. Census Region: ^c^			
Midwest	438 (24.9)	5.3 (4.8, 5.8)	8,207,633 (16.9)
Northeast	206 (11.7)	4.2 (3.6, 4.8)	4,920,495 (10.2)
South	381 (21.7)	2.4 (2.2, 2.7)	15,575,099 (32.1)
West	268 (15.3)	3.5 (3.1, 3.9)	7,681,621 (15.9)
PR/VI	2 (0.1)	--^a^	374,127 (1)
			*N* = 36,758,975

*Note*: AI/AN, American Indian/Alaskan Native; NH/OPI, Native Hawaiian/Other Pacific Islander; PR/VI, Puerto Rico/Virgin Islands

^a^CI not calculated for cell size < 10

^b^Denominator data for race/ethnicity were available for 2013–2017 only, therefore rates are based on data for these years only

^c^Denominator data for U.S. Census region were available for 2012–2017 only, therefore rates are based on data for these years only

Compared to suspected cases, confirmed/probable cases were more significantly associated with intentional self-harm and undetermined causes of injury, whereas suspected cases were more likely to be coded as unintentional (*p* <  0.001 for each injury category/case classification combination except assault) (Table 1). Significant differences were also seen with race/ethnicity of confirmed/probable cases compared to suspected with more confirmed/probable cases being white or multi-racial (*p* = 0.001 and 0.01, respectively). Confirmed/probable cases were significantly more likely to receive care in inpatient settings (26.1% confirmed/probable versus 7.4% for suspected cases; *p* <  0.001). Additionally, 614 of 1274 (48.2%) confirmed/probable cases seen in the outpatient setting were ED or urgent care visits.

EMR review of 595 patients initially classified as confirmed/probable CO poisoning with undetermined injury intent revealed that 310 (52.1%) were unintentional CO poisoning. Intentional self-harm accounted for 27 (4.5%) while injury intent for CO poisoning for the remaining 258 (43.4%) could not be determined. An additional review of 20 randomly selected EMR of confirmed/probable cases with source of CO poisoning “other or unspecified” revealed that 5 (25%) in fact had a motor vehicle exhaust source. The largest percent (7 of 20 cases; 35%) remained with source other or unspecified. Other sources were 3 smoke/fumes (15%), 2 each utility gas and liquid propane (10% each), and 1 incomplete combustion of domestic fuel (5%).

Among suspected cases, ICD-9-CM code E825.x, “other motor vehicle non-traffic accident of other and unspecified nature, including accidental poisoning from carbon monoxide” was present most frequently (49.4%) followed by E869.9, “accidental poisoning by other gases or vapors, unspecified,” (19.2%). Review of 20 randomly selected EMR of patients coded with E825.x revealed that all were involved in some type of fall from, or non-collision event involving a motor vehicle (most commonly, a motorcycle); none had evidence of CO poisoning.

**Fig. 2:**
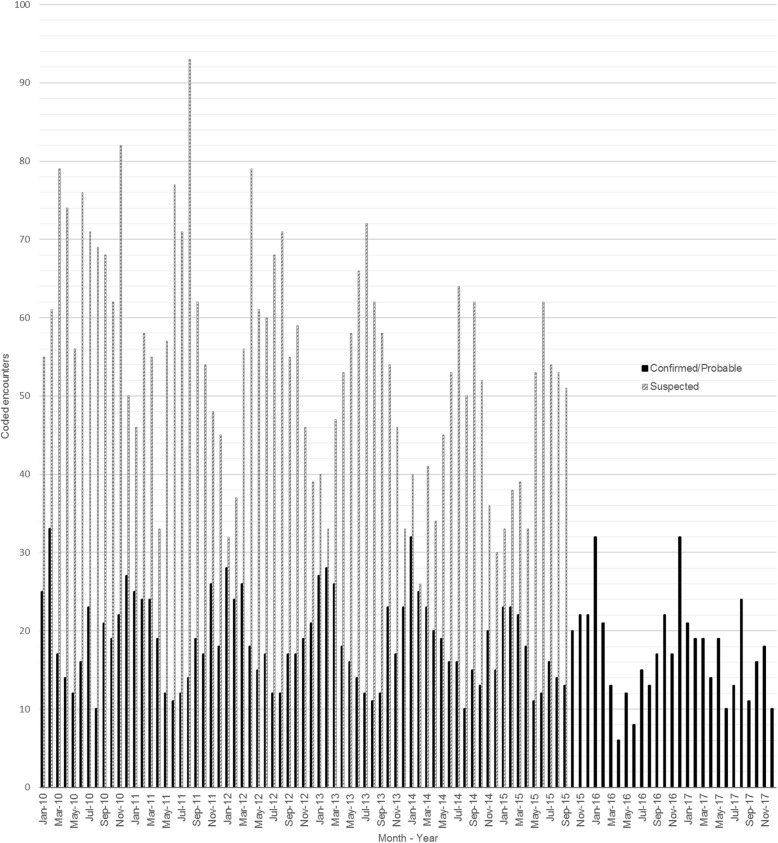
Confirmed/probable and suspected carbon monoxide poisoning unique encounters, showing comparative seasonality, 2010-2017. Note: Suspected cases (based on ICD-9-CM codes only) did not occur after September 30, 2015 when coding system converted to ICD-10-CM

CO poisoning encounters demonstrated a distinct seasonal pattern, with confirmed/probable cases peaking in winter months, and decreasing in summer (Fig. 2). Two peaks, occurring in July 2010 and August 2017 vary from this pattern. However, confirmed/probable cases during these months occurred at multiple facilities across multiple states and could not be linked to specific natural disaster events. Similarly, we detected no geographic clustering of cases within seasonal peaks linked to specific events. Suspected CO poisoning encounters show a reverse seasonal pattern, with occurrence, particularly those with code E825.x, tending to peak in summer months and nadir in the winter. Since the change from ICD-9-CM to ICD-10-CM systems, overall numbers of coded cases of CO poisoning (primarily suspected cases) appear to have dropped, with categories shifting toward more unintentional and fewer intentional self-harm and undetermined cases.

Percent COHb blood level measurement was performed at the time of CO poisoning encounter in 850 out of 5491 cases (15.5%) (Table 2). COHb blood level measurement occurred more frequently among confirmed/probable cases (38%), than among suspected (4.9%) (*p* <  0.001). A COHb level > 9% was found in 30.8% of confirmed/probable cases, compared to 4.4% of patients with suspected CO poisoning (*p* <  0.001).

Seventeen (1%) patients with confirmed/probable CO poisoning had HBO_2_ therapy based on procedure codes. No suspected CO poisoning cases had documented HBO_2_ therapy. All HBO_2_ treatments were performed at non-VA facilities, as VHA facilities don’t provide HBO_2_ treatment onsite. Percent COHb blood level for 15 of 17 patients at the time of CO exposure ranged between 16 and 50% (median 27.6%).

More patients with confirmed/probable compared to suspected CO poisoning died within 30 days and within 1 year after their encounter (1.5 and 5.1% at 30-day and 1-year intervals for confirmed/probable versus 0.1 and 0.3% for suspected at 30-day and 1-year intervals, respectively; *p* <  0.001 in both cases). Table 3 lists all confirmed/probable cases who died within 30 days of initial CO poisoning encounter, and their cause of death. Of 26 (1.5%) confirmed/probable cases who died within 30-day time frame, ages ranged from 30 to 85 years (median 63). Causes of death were primarily cardiovascular (11 of 26 (42%) cases), including cardiac arrest and congestive heart failure in 3 cases each, ST-elevation myocardial infarction and inferior wall myocardial infarction in 2 cases each, and 1 stroke). Anoxic brain injury occurred in 4 of 26 (15%) cases, one of whom was unsuccessfully treated with HBO_2_ after CO poisoning exposure during a house fire. Repeat successful suicide attempt by more lethal means (following original unsuccessful attempt using CO), lung disease, and cases whose EMR documentation was inadequate to allow determination of cause of death followed with 3 each (3 of 26 (12%) for each category). Acute kidney injury was least common with 2 of 26 (8%) cases.

**Table 3:** Causes of death for carbon monoxide poisoning cases who died within 30 days of encounter, 2010–2017

Age	# Days, encounter until death	ICD Exposure Code	Code description; injury category	Cause of death	Category of death
60	1	E890.2	Other smoke and fumes from conflagration in a private dwelling, including carbon monoxide; unintentional	Anoxic brain injury	Anoxic brain injury
30	0	T58.02X	Toxic effect of carbon monoxide from motor vehicle exhaust; intentional self-harm	Death by suicide (original attempt)	Anoxic brain injury
62	25	986	Toxic effect of carbon monoxide; undetermined	Anoxic brain injury	Anoxic brain injury
78	28	986	Toxic effect of carbon monoxide; undetermined	Anoxic brain injury	Anoxic brain injury
63	8	E868.8	Accidental poisoning by carbon monoxide from other sources; unintentional	STEMI (ST - elevation myocardial infarction)	Cardiovascular
70	21	T58.01X	Toxic effect of carbon monoxide from motor vehicle exhaust; unintentional	Congestive heart failure	Cardiovascular
81	3	T58.8X1	Toxic effect of carbon monoxide from other source; unintentional	Myocardial infarction	Cardiovascular
67	8	T58.91X	Toxic effect of carbon monoxide from unspecified source; unintentional	STEMI (ST - elevation myocardial infarction)	Cardiovascular
63	1	986	Toxic effect of carbon monoxide; undetermined	Inferior wall myocardial infarction	Cardiovascular
61	2	986	Toxic effect of carbon monoxide; undetermined	Cardiac arrest	Cardiovascular
67	8	986	Toxic effect of carbon monoxide; undetermined	Congestive heart failure	Cardiovascular
38	10	986	Toxic effect of carbon monoxide; undetermined	Cardiac arrest	Cardiovascular
63	13	986	Toxic effect of carbon monoxide; undetermined	Stroke	Cardiovascular
66	19	986	Toxic effect of carbon monoxide; undetermined	Congestive heart failure	Cardiovascular
53	30	986	Toxic effect of carbon monoxide; undetermined	Cardiac arrest	Cardiovascular
85	0	E868.9	Accidental poisoning by carbon monoxide from unspecified source; unintentional	Acute kidney injury	Kidney injury
69	6	T58.11X	Toxic effect of carbon monoxide from utility gas; unintentional	Acute kidney injury	Kidney injury
68	11	986	Toxic effect of carbon monoxide; undetermined	Lung cancer	Lung disease
63	24	986	Toxic effect of carbon monoxide; undetermined	End-stage COPD	Lung disease
63	25	986	Toxic effect of carbon monoxide; undetermined	End-stage COPD	Lung disease
52	21	E952.1	Self-inflicted poisoning by other carbon monoxide source; intentional self-harm	Self-inflicted poisoning, carbon monoxide	Suicide - repeat attempt
43	27	E952.1	Self-inflicted poisoning by other carbon monoxide source; intentional self-harm	Death by suicide (new attempt, different method)	Suicide - repeat attempt
57	9	T58.92X	Toxic effect of carbon monoxide from unspecified source; intentional self-harm	Death by suicide (new attempt, different method)	Suicide - repeat attempt
60	18	E868.9	Accidental poisoning by carbon monoxide from an unspecified source; unintentional	Unknown (inadequate chart documentation)	Unknown
63	26	E868.1	Accidental poisoning by other/unspecified utility gas, or carbon monoxide from combustion of such gas; unintentional	Unknown (inadequate chart documentation)	Unknown
70	8	T58.91X	Toxic effect of carbon monoxide from unspecified source; unintentional	Unknown (inadequate chart documentation)	Unknown

## Discussion

We determined that from 2010 through 2017, the average annual rate for CO poisoning among Veterans was 3.6 confirmed/probable cases per 100,000 unique users of VHA care. Our study focused on Veterans receiving care in VHA and does not exclude fire-related or intentional CO poisoning exposures, therefore the annual rate of confirmed/probable CO cases must be viewed in that context when compared to national UNFR rates based on U.S population estimates. Nevertheless, some useful comparisons can be made.

We detected significant differences between confirmed/probable and suspected CO poisoning cases, indicating that confirmed/probable are likely more severely affected (i.e. presence of COHb blood level > 9%, more frequent inpatient encounters, treatment with HBO_2_, increased frequency of death within 30 days or 1 year of initial encounter) than cases classified as suspected. We found that confirmed/probable cases were more likely to be associated with intentional self-harm and undetermined injury categories. However, in our review of a subset of 595 confirmed/probable cases with undetermined injury intent, 53% represented unintentional cases, so the unintentional category of injury may have been underrepresented among our confirmed/probable CO poisoning cases. Underreporting of CO poisoning source also occurred, however our review of 20 randomly selected EMR of confirmed/probable cases with source of CO poisoning “other or unspecified” revealed occurrence of sources comparable to those of the complete cohort.

Our demographic trends for confirmed/probable CO poisoning were consistent with some previous studies, with younger age groups being more affected [1, 24, 25], and location in Midwest U.S. Census regions [1, 26, 27]. Although other studies reported higher rates among older age groups and females, we did not see this increase in our study. Our analysis showing association of confirmed/probable CO poisoning cases with self-harm, and the increased incidence of suicide among younger Veterans [15] may partially explain this finding. However, differing methodologies such as exclusion of intentional and non-fire-related injuries, and inclusion of mortality data may make comparisons with these studies problematic [26, 28].

Confirmed/probable cases followed the established cold weather seasonal trends commonly associated with CO poisoning [24, 29], while suspected cases followed a distinctly opposite trend. The summer seasonal trend shown by the suspected CO poisoning cases may be explained by the relatively high frequency of the E825.x code, which our EMR review indicated may be more indicative of motorcycle and other motor vehicle type-associated non-collision events than CO poisoning. These events could be expected to peak during summer months [30]. If our EMR review of 20 encounters with an E825.x code was representative of all 1847 cases classified as such, then approximately half of the suspected cases in our cohort were likely not true cases. The transition in October 2015 from ICD-9-CM to ICD-10-CM effectively removed the suspected CO poisoning category from consideration going forward within VHA. Findings like these in which disease surveillance trends shift as a result of coding differences before and after the U.S. transition from ICD-9-CM to ICD-10-CM are not unusual, and can make year-to-year comparisons challenging [31].

Studies showing elevated risk of short-term and long-term mortality after CO poisoning, especially with respect to adverse cardiovascular events, shared similarity with our results [14, 32–34]. One concerning finding of our study was that of 3 patients who attempted suicide using CO, they successfully repeated their attempt within 30 days, using more lethal means. Given the increased rates of suicide among Veterans, VHA has targeted for improvement specific methods to follow-up and prevent suicide in patients deemed high-risk [35].

### Limitations

Our study has several limitations. In limiting cases to those coded with ICD-9-CM or ICD-10-CM CO poisoning codes, we may have omitted those miscoded or not coded. In addition, clinicians may not have considered CO poisoning when patients presented to the ED. Previous studied have found that 3–5% of patients presenting with headache and dizziness had occult CO poisoning [36], and therefore could have contributed to fewer cases found in our study. We did not include National Death Index data, so patients who died from their CO poisoning before receiving treatment at a VHA hospital are not included in our study. While we attempted to limit inclusion of miscoded cases caused by carry-over of historic exposure codes, we cannot be sure that all cases represented current diagnoses of CO poisoning. Conversely, our method of limiting cases to initial occurrence only may have eliminated cases that represented true second exposures, for example, repeat suicide attempts or unintentional exposures in homes where original source of CO has not been remediated. Our review of cases classified as “undetermined” indicates misuse of this category code may have led to underrepresentation of unintentional and intentional injury intent categories of CO poisoning in our study. Similarly, we found a high percentage of cases coded with an “other or unspecified” source of CO poisoning. Our evaluation of COHb blood values was likely limited by the fact that initial exposure to CO is often measured at the scene of CO exposure by first responders using pulse CO-oximetry. These values, and those of patients transferred to non-VA emergency care are scanned into the Veterans’ EMR and thus not available as discrete, electronically queriable LOINC codes. Our review of the 17 cases treated with HBO_2_ indicates that COHb levels at time of treatment and recorded within scanned documents were much higher than measurements available in the EMR for those patients. Capture of HBO_2_ treatment may be similarly limited as it is routinely performed at non-VA sites.

## Conclusions

CO poisoning is important for targeted public health surveillance in VHA. Despite the pitfalls of an administrative, ICD code-based surveillance system, its advantages are that it can be implemented nationally with relatively few resources, and that it provides useful, actionable information. Improvements are needed in ICD coding to reduce the percentage of cases coded with unknown injury intent and/or CO poisoning source. Opportunities exist to implement VHA-wide provider education to better recognize signs, symptoms, and exposure history for CO poisoning; and patient education programs addressing identified areas of high-risk for CO poisoning among Veterans, augmenting the VA Disaster Preparedness Manual, which contains useful information on preventing exposure to CO. [37] Examination of CO poisoning suicide attempts to prevent progression to more lethal means may be an area of further study.

## Additional file

Additional file 1: **Table S1.** Occurrence of ICD-9-CM and ICD-10-CM codes for classification of carbon monoxide poisoning cases among Veterans, 2010-2017^a^ (DOCX 21 kb)

## Abbreviations

CO: Carbon monoxide
COHb: carboxyhemoglobin
CSTE: Council of State and Territorial Epidemiologists
ED: emergency department
EMR: electronic medical record
HBO_2_: hyperbaric oxygen
ICD: International Classification of Diseases
ICD-10-CM: International Classification of Diseases, 10th revision, Clinical Modification
ICD-9-CM: International Classification of Diseases, 9th revision, Clinical Modification
UNFR: unintentional, non-fire related
VA: Department of Veterans Affairs
VHA: Veterans Health Administration
