# Supplementary material for: Carbon monoxide poisoning surveillance in the Veterans Health Administration, 2010–2017
Source: BMC Public Health. 2019 Feb 14;19:190. doi: 10.1186/s12889-019-6505-y (PMC6376743; doi:10.1186/s12889-019-6505-y)
Supplement: Supplementary file 1 — Table S1. Occurrence of ICD-9-CM and ICD-10-CM codes for classification of carbon monoxide poisoning cases among Veterans, 2010-2017a (DOCX 21 kb) [file 12889_2019_6505_MOESM1_ESM.docx]

**Additional file 1: Table S1. Occurrence of ICD-9-CM and ICD-10-CM codes for classification of carbon monoxide poisoning cases among Veterans, 2010-2017^a^**

| ICD 9/10 Code | Confirmed/ probable, N = 1,755 (%) | Suspected, N = 3,736 (%) | ICD 9/10 Description (poisoning source) | Injury Category |
| --- | --- | --- | --- | --- |
| E868.3 | 41 (2.3) | 0 | Accidental poisoning by carbon monoxide from incomplete combustion of other domestic fuels | Unintentional |
| E868.8 | 73 (4.2) | 0 | Accidental poisoning by carbon monoxide from other sources | Unintentional |
| E868.9 | 274 (15.6) | 0 | Accidental poisoning by carbon monoxide from unspecified source | Unintentional |
| E868.2 | 40 (2.3) | 0 | Accidental poisoning by motor vehicle exhaust gas not elsewhere classifiable | Unintentional |
| E818.x | 0 | 0 | Other non-collision motor vehicle traffic accident, including accidental poisoning from exhaust gas | Unintentional |
| E825.x | 2 (0.1) | 1,847 (49.4) | Other motor vehicle non-traffic accident of other and unspecified nature, including accidental poisoning from carbon monoxide | Unintentional |
| E838.x | 0 | 193 (5.2) | Other and unspecified water transport accident, including accidental poisoning by bases or fumes on ship | Unintentional |
| E844.x | 0 | 273 (7.3) | Other specified air transport accidents, including poisoning by carbon monoxide while in transit | Unintentional |
| E867 | 2 (0.1) | 0 | Accidental poisoning by gas distributed by pipeline, or carbon monoxide from combustion of such gas | Unintentional |
| E868.0 | 4 (0.2) | 9 (0.2) | Accidental poisoning by liquefied petroleum gas (LPG) in mobile containers, or carbon monoxide from combustion of such gas | Unintentional |
| E868.1 | 8 (0.5) | 40 (1.1) | Accidental poisoning by other/unspecified utility gas, or carbon monoxide from combustion of such gas | Unintentional |
| E869.9 | 14 (0.8) | 717 (19.2) | Accidental poisoning by other gases or vapors, unspecified | Unintentional |
| E890.2 | 7 (0.7) | 104 (2.8) | Other smoke and fumes from conflagration in a private dwelling, including carbon monoxide | Unintentional |
| E891.2 | 0 | 76 (2.0) | Other smoke and fumes from conflagration in other building, including carbon monoxide | Unintentional |
| E962.2 | 3 (0.2) | 4 (0.1) | Homicidal assault by poisoning from other gases and vapors | Assault |
| E962.9 | 0 | 6 (0.2) | Homicidal assault by poisoning, unspecified | Assault |
| E968.0 | 0 | 9 (0.2) | Homicidal assault by fire | Assault |
| E952.1 | 199 (11.3) | 0 | Self-inflicted poisoning by other carbon monoxide source | Intentional Self-harm |
| E952.0 | 97 (5.5) | 0 | Self-inflicted poisoning by motor vehicle exhaust gas | Intentional Self-harm |
| E951.0 | 1 (0.1) | 3 (0.1) | Self-inflicted poisoning by gases in domestic use, pipeline | Intentional Self-harm |
| E951.1 | 1 (0.1) | 4 (0.1) | Self-inflicted poisoning by gases in domestic use, LPG (mobile) | Intentional Self-harm |
| E951.8 | 1 (0.1) | 29 (0.8) | Self-inflicted poisoning by gases in domestic use, other utility gas | Intentional Self-harm |
| E952.9 | 0 | 17 (0.5) | Self-inflicted poisoning by other gases or vapors, unspecified | Intentional Self-harm |
| E958.1 | 0 | 169 (4.5) | Self-inflicted injury by burns, fire | Intentional Self-harm |
| E982.1 | 26 (1.5) | 0 | Undetermined cause of poisoning by other carbon monoxide source | Undetermined |
| 986 | 492 (28.0) | 0 | Toxic effect of carbon monoxide | Undetermined |
| E982.0 | 2 (0.1) | 0 | Undetermined cause of poisoning by motor vehicle exhaust gas | Undetermined |
| E972, E978 | 0 | 0 | Legal intervention of execution including asphyxiation by gas | Undetermined |
| E979.3 | 0 | 8 (0.2) | Terrorism involving fires, including asphyxia | Undetermined |
| E981.0 | 0 | 0 | Poisoning by gases in domestic use, undetermined intent, pipeline | Undetermined |
| E981.1 | 1 (0.1) | 0 | Poisoning by gases in domestic use, undetermined intent, LPG (mobile) | Undetermined |
| E981.8 | 0 | 5 (0.1) | Poisoning by gases in domestic use, undetermined intent, other utility gas | Undetermined |
| E988.1 | 0 | 196 (5.2) | Undetermined cause of injury by fire, burns | Undetermined |
| 987 | 0 | 27 (0.2) | Toxic effect of other gases, fumes or vapors | Undetermined |
| T58.01 | 73 (4.2) | 0 | Toxic effect of carbon monoxide from motor vehicle exhaust, accidental (unintentional) | Unintentional |
| T58.11 | 36 (2.1) | 0 | Toxic effect of carbon monoxide from utility gas, accidental (unintentional) | Unintentional |
| T58.2X1 | 52 (3.0) | 0 | Toxic effect of carbon monoxide from incomplete combustion of other domestic fuels, accidental (unintentional) | Unintentional |
| T58.8X1 | 62 (3.5) | 0 | Toxic effect of carbon monoxide from other source, accidental (unintentional) | Unintentional |
| T58.91 | 58 (3.3) | 0 | Toxic effect of carbon monoxide from unspecified source, accidental (unintentional) | Unintentional |
| T58.03 | 3 (0.2) | 0 | Toxic effect of carbon monoxide from motor vehicle exhaust, assault | Assault |
| T58.13 | 3 (0.2) | 0 | Toxic effect of carbon monoxide from utility gas, assault | Assault |
| T58.2X3 | 0 | 0 | Toxic effect of carbon monoxide from incomplete combustion of other domestic fuels, assault | Assault |
| T58.8X3 | 1 (0.1) | 0 | Toxic effect of carbon monoxide from other source, assault | Assault |
| T58.93 | 0 | 0 | Toxic effect of carbon monoxide from unspecified source, assault | Assault |
| T58.02 | 34 (1.9) | 0 | Toxic effect of carbon monoxide from motor vehicle exhaust, intentional self-harm | Intentional Self-harm |
| T58.12 | 2 (0.1) | 0 | Toxic effect of carbon monoxide from utility gas, intentional self-harm | Intentional Self-harm |
| T58.2X2 | 3 (0.2) | 0 | Toxic effect of carbon monoxide from incomplete combustion of other domestic fuels, intentional self-harm | Intentional Self-harm |
| T58.8X2 | 4 (0.2) | 0 | Toxic effect of carbon monoxide from other source, intentional self-harm | Intentional Self-harm |
| T58.92 | 7 (0.4) | 0 | Toxic effect of carbon monoxide from unspecified source, intentional self-harm | Intentional Self-harm |
| T58 | 0 | 0 | Toxic effect of carbon monoxide | Undetermined |
| T58.0 | 0 | 0 | Toxic effect of carbon monoxide from motor vehicle exhaust | Undetermined |
| T58.04 | 12 (0.7) | 0 | Toxic effect of carbon monoxide from motor vehicle exhaust, undetermined | Undetermined |
| T58.1 | 0 | 0 | Toxic effect of carbon monoxide from utility gas | Undetermined |
| T58.14 | 19 (1.1) | 0 | Toxic effect of carbon monoxide from utility gas, undetermined | Undetermined |
| T58.2 | 0 | 0 | Toxic effect of carbon monoxide from incomplete combustion of other domestic fuels | Undetermined |
| T58.2X | 0 | 0 | Toxic effect of carbon monoxide from incomplete combustion of other domestic fuels | Undetermined |
| T58.2X4 | 5 (0.3) | 0 | Toxic effect of carbon monoxide from incomplete combustion of other domestic fuels, undetermined | Undetermined |
| T58.8 | 0 | 0 | Toxic effect of carbon monoxide from other source | Undetermined |
| T58.8X | 0 | 0 | Toxic effect of carbon monoxide from other source | Undetermined |
| T58.8X4 | 64 (3.6) | 0 | Toxic effect of carbon monoxide from other source, undetermined | Undetermined |
| T58.9 | 0 | 0 | Toxic effect of carbon monoxide from unspecified source | Undetermined |
| T58.94X | 29 (1.7) | 0 | Toxic effect of carbon monoxide from unspecified source, undetermined | Undetermined |

^a^For each unique carbon monoxide poisoning case, the most descriptive ICD-9-CD or ICD-10-CM code (i.e., the code used to categorize the case as unintentional, intentional self-harm, assault, or undetermined) is shown in the table. For example, if a confirmed case was coded with ICD-9-CM code 986, “toxic effect of carbon monoxide” and the cause of injury code E867, “Accidental poisoning by gas distributed by pipeline, or carbon monoxide while in transit,” code E867 is indicated in the confirmed/probable column as the most descriptive code present for this patient.
